# A Few Verifications

**Published:** 1888-11

**Authors:** H. G. Glover

**Affiliations:** Jackson, Mich.


### A FEW VERIFICATIONS.

H. G. Glover, M. D., Jackson, Mich.

Case I.—June 2d.—George A. consulted me for a toothache which had been "going it" all the week. He said if I would save him the tooth (the only molar left on right side of lower jaw), he would make me a nice present. With such an unusual offer for a doctor to receive, how could I fail to do my best?

I could get but few reliable symptoms, but found he was worse after going to bed, tooth loose. Temple, cheek, and jaw sore to touch. Gave Merc. sol. 3d and 4th, powders, Placebo to follow. The next evening I saw him again; no better. On carefully reviewing the case I found Nux vom. strongly indicated by the concomitants. Gave three powders of the 1m. The next evening he came in with the remark, "Doctor, you have won your present." The toothache had departed. To my inquiry as to how he had slept the previous night, he said, "Better than for two weeks." So much for the indicated remedy.

Case II.—June 9th.—Frank T. came into my office, suffering with a severe pain in the lumbar region, which had taken him suddenly the evening before, and had proceeded to make "the night hideous" for him.

Diagnosis.—Lumbago; remedy, Rhus tox., as indicated by the characteristic aggravation and amelioration. Gave Rhus $^{cc}$ three powders. A few days ago I met him, and he said that he felt better after taking the first powder, and that the pain was gone by evening, and he had a good night's rest. With some comments on the size of my cranium, he bade me "good-day."

CASE III.—June 8th.—I was called to see what looked very much like a case of diphtheria in its incipiency, in the little son of an old family of Dr. J. W. Dowling's, who were strangers here, but *would* have a homœopath if there was one in the city. Right tonsil inflamed, swollen, with a grayish-yellow patch about the size of a dime; tongue heavily coated; pulse 110; agg. four P. M. to bed-time; rumbling in the bowels. Of course, I gave Lyc.$^{1m}$, two powders. Next morning tonsil cleared off; better every way. Left Sac. Lac. with instructions to let me know if further attention seemed necessary. Mother came in June 15th and reported the boy all right. We are having a severe epidemic of diphtheria here at present, which makes it seem highly probable that Lyc. "knocked out" a case there in the "first round."

These cases illustrate very nicely, I think, the efficacy of *the* indicated remedy and the minimum dose.

Homœopathy is an exact science which few men ever master, but we can all do good work if we only "Learn to labor and not wait."

